# Whole-Exome Sequencing Reveals a Rare Missense Variant in *SLC16A9* in a Pedigree with Early-Onset Gout

**DOI:** 10.1155/2020/4321419

**Published:** 2020-01-31

**Authors:** Xiu-Feng Huang, Li Sun, Chunwu Zhang, Zhenni Zhou, Hui Chen, Linhua Zhang, Matthew A. Brown, Xiaoru Xia

**Affiliations:** ^1^Institute of Health and Biomedical Innovation, Queensland University of Technology, Translational Research Institute, Brisbane, QLD, Australia; ^2^Department of Rheumatology, First Affiliated Hospital of Wenzhou Medical University, Wenzhou, China; ^3^Department of Injury Orthopaedics, First Affiliated Hospital of Wenzhou Medical University, Wenzhou, China; ^4^Department of Internal Medicine, Yueqing People's Hospital, Yueqing, China; ^5^Department of Nephrology, Wenzhou Central Hospital, Wenzhou, China; ^6^Department of Clinical Laboratory, Yuhuan People's Hospital, Yuhuan, China; ^7^Centre for Precision Medicine, First Affiliated Hospital of Wenzhou Medical University, Wenzhou, China; ^8^Guy's & St Thomas' NHS Foundation Trust and King's College London NIHR Biomedical Research Centre, London, UK

## Abstract

Gout is a common inflammatory arthritis triggered by monosodium urate deposition after longstanding hyperuricemia. In the general community, the disease is largely polygenic in genetic architecture, with many polymorphisms having been identified in gout or urate-associated traits. In a small proportion of cases, rare high penetrant mutations associated with monogenic segregation of the disease in families have been demonstrated to be disease causative. In this study, we recruited a two-generation pedigree with early-onset gout. To elucidate the genetic predisposition, whole-exome sequencing (WES) was performed. After comprehensive variant analyses and cosegregation testing, we identified a missense variant (c.277C>A, p.L93M) in *SLC16A9*, an extremely rare variant in genetic databases. Moreover, in silico assessments showed strong pathogenicity. This variant cosegregated with the disease phenotype perfectly in the family and is located in a highly conserved functional domain. A few studies supported our results of the association between *SLC16A9* and gout and serum urate levels. In conclusion, we provide the first evidence for the association of rare missense in *SLC16A9* with early-onset gout. These findings not only expand our current understanding of gout but also may have further implications for the treatment and prevention of gout.

## 1. Introduction

Gout is a common inflammatory arthritis caused by the deposition of monosodium urate (MSU) crystals in and around the joints following longstanding hyperuricemia [1]. It affects 1-2% of adults in developed countries [1–3] and has a prevalence of 1.14% in eastern China [4]. Similar to other complex phenotypes, gout results from the interplay between inherited genetic risk variants and environmental factors [5]. Genome-wide association studies (GWAS) have confirmed the importance of genetic basis in gout. Several genetic loci have been associated with gout, such as *ABCG2*, *ALDH16A1*, *BCAS3*, *RFX3*, *KCNQ1*, *ATXN2*, *CUX2*, *GCKR*, *PDZK1*, *CNTN5*, and mitochondrial genetic variation [6–14]. For example, ABCG2 dysfunctional variants have a strong impact on the progression of hyperuricemia. The most common dysfunction variant rs2231142 (p.Q141K) increases the risk of gout and hyperuricemia, significantly influences the age of onset of gout, and is highly associated with a familial gout history [15]. Moreover, ABCG2 dysfunction was reported as a strong independent risk factor for pediatric-onset hyperuricemia/gout [16].

Notably, almost all these loci identified in gout GWAS were also associated with serum urate levels, indicating the shared genetic basis between gout and serum urate concentrations [5]. This is mainly because elevated serum urate levels are a critical risk factor for gout onset [17]. However, as GWAS for gout have been relatively limited in size and power compared with the GWAS of serum urate levels, less is known about the specific genetic contribution to gout as opposed to genetic associations of hyperuricemia. Association with gout at most urate-associated loci is still unclear.

*SLC16A9* encodes monocarboxylate transporter 9 (MCT9), a member of solute carrier (SLC) superfamily that comprises more than 400 transporters [18]. *SLC16A9* is ubiquitously expressed, including at particularly high levels in the kidney [19, 20]. Recent GWAS and meta-analysis revealed a significant association between polymorphisms of *SLC16A9* and serum urate concentrations [7, 21, 22]. However, a role for variants of *SLC16A9* and gout itself has not been demonstrated [7, 11]. To date, the transport substrate of MCT9 is still unknown and the function of *SLC16A9* remains poorly understood, especially its potential association with gout.

Despite dozens of genetic loci identified in gout or urate-associated traits, little is known about the genetic aetiology of patients presenting with early-onset gout (EOG), which was defined as before the age of 40 years [23–26]. Previous studies have reported rs2231142 (Q141K) in *ABCG2* as a genetic factor in early-onset gout [16, 27, 28]. In this study, we investigated a two-generation pedigree with early-onset gout. To elucidate the genetic predisposition, whole-exome sequencing (WES) was performed, and we identified a rare missense mutation (c.277C>A, p.L93M) in *SLC16A9*, providing new evidence for the association of *SLC16A9* with gout.

## 2. Materials and Methods

### 2.1. Participant Recruitment

This study conformed to the tenets of the Declaration of Helsinki and was approved by the Ethics Committee of the First Affiliated Hospital of Wenzhou Medical University. Informed consent was obtained from the patient. Patients were clinically evaluated by rheumatologist according to the 2015 gout classification criteria by an American College of Rheumatology/European League against Rheumatism Collaborative Initiative [29, 30]. Peripheral blood samples were collected from patients and unaffected family members, from which genomic DNA was extracted.

### 2.2. Whole-Exome Sequencing

Whole-exome sequencing (WES) was performed on the proband. Briefly, genomic DNA was sheared into 200- to 250-base pair (bp) fragments using a Covaris S220 ultrasonicator. Then the fragments were ligated with adapters to both ends, amplified by ligation-mediated polymerase chain reaction, purified, and hybridized. Nonhybridized fragments were washed out. Enrichment of the DNA libraries was performed using the Exome Enrichment V5 Kit (Agilent Technologies, Palo Alto, CA, USA) according to the manufacturers' protocol. Subsequently, enriched DNA libraries were sequenced on a HiSeq X Ten sequencer (Illumina, San Diego, CA, USA). All raw sequencing data were processed according to a customized bioinformatics pipeline described previously [31]. After the quality control test, the reads were mapped to the reference human genome (hg19) using SOAPaligner software and further visualized using the SplicingViewer software [32]. SNV and Indel calls as well as annotation were performed using GATK tool and mirTrios with integrated ANNOVAR tool [33].

### 2.3. Variant Analyses and Identification

We used the following databases for selecting rare variants as an initial filtration: Genome Aggregation Database (http://gnomad.broadinstitute.org/), Exome Aggregation Consortium (ExAC, http://exac.broadinstitute.org/), NHLBI Exome Sequencing Project (ESP, http://evs.gs.washington.edu/EVS/), and 1000 Genome (http://www.1000genomes.org). Variants with a minor allele frequency of over 0.01 in any of these databases were discarded. The effects of the candidate variants were assessed using in silico prediction programs. Missense variants were analyzed by M-CAP (http://bejerano.stanford.edu/mcap/), Polyphen-2 (http://genetics.bwh.harvard.edu/pph2/), and MutationTaster (http://mutationtaster.org/). Direct Sanger sequencing was then used to confirm the segregated variants in the present family, using an ABI 3500 Genetic Analyzer (Applied Biosystems, Carlsbad, CA, USA).

### 2.4. *SLC16A9* Amplification and Genotyping

An additional cohort of unrelated cases (*n* = 30) with gout was recruited, and their DNA was submitted for Sanger sequencing. Primers were designed to amplify all coding regions and the intron-exon boundaries of the *SLC16A9* gene. The PCR products were purified and sequenced on an ABI 3500 Genetic Analyzer (Applied Biosystems, Carlsbad, CA, USA).

## 3. Results

### 3.1. Clinical Observations

**Figure 1:**
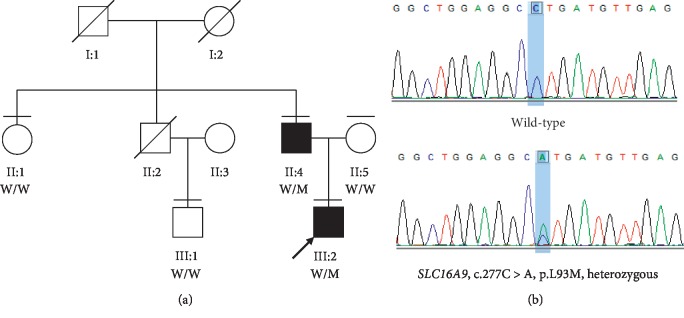
Identification of SLC16A9 missense in the family with early-onset gout. (a) Pedigree and cosegregation results. Affected individual is represented as a filled square. Normal individuals are shown as empty symbols. (b) Sanger sequencing confirmed the segregation of the rare missense variant, c.277C>A (p.L93M).

The proband was a 25-year-old male from a Han Chinese family, having suffered his first gout flare at the age of 20. His father was also diagnosed with gout having the first gout flare at the age of 25, whereas all the other family members were unaffected (Table 1 and Figure 1(a)).

Both proband and his affected father experienced recurrent acute monoarticular arthritis affecting the first metatarsophalangeal joint (MTP1) and/or knee starting at 20 and 25 years of age, respectively. The symptoms generally started at night and peaked within 24 hours, preventing walking and could not bear touch. The symptomatic course lasted no more than one week. The symptoms typically completely resolved within one or two days after taking nonsteroidal anti-inflammatory drug and colchicine. Patients have normal intelligence and are competent of the job. The muscle tension and renal function are normal, and no urate nephrolithiasis has been found. In both cases, serum uric acid was increased but not achieved to an extremely high level (see in Table 1), and the fractional excretion of uric acid (FEUa) was decreased (the normal range for FEUa is 7%–12%) [30, 34], consistent with renal underexcretion (RUE) gout. So the purine overproduction gout (HGPRT deficiency, PRPS1 superactivity) was excluded in the family. The detailed clinical information is summarized in Table 1.

### 3.2. Genetic Assessments

**Figure 2:**
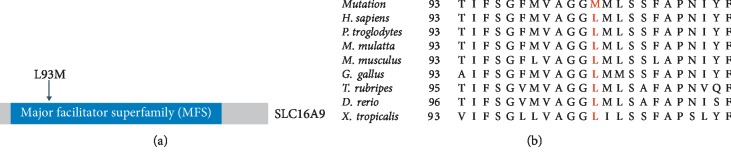
(a) Domain structure of SLC16A9 and location of L93M variant. (b) Conservation analyses of the mutated residues 93 in *SLC16A9* across different species.

To reveal the genetic predisposition, WES was performed on the proband (III:2). The mean read depth for the WES was >100X and the coverage of the targeted regions (>1X) reached >99%. Variant analyses and a step-by-step filtering strategy by combination of minor allele frequency, in silico assessments, gene function, and cosegregation analysis were carried out [35–37]. A total of six candidate variants were submitted for cosegregation analysis and only one survived, a rare missense mutation (c.277C>A, p.L93M) in *SLC16A9*. This is an extremely rare variant (rs550527563) in all of the databases (Table 2). For example, the allele frequency is 0.0032% (8 in 251050) and 0.0033% (4 in 120986) in gnomAD and ExAC, respectively, while it is absent in ESP (Table 2). All these alleles are from Asian, while it is absent in Caucasians. Moreover, in silico assessments showed strong pathogenicity for this variant including the M-CAP, a newly developed tool for variants with uncertain significance in clinical exomes at high sensitivity [38]. Importantly, segregation testing in all available family members indicated that L93M cosegregated with the disease phenotype in this pedigree (Figures 1(a) and 1(b)). Both patients harbor a heterozygous variant while the healthy individuals do not have the nucleotide change. The variant c.277C>A results in a switch from leucine to methionine in the major facilitator superfamily (MFS) domain (Figure 2(a)). Multiple orthologous sequence alignment revealed that leucine at position 93 is in a highly conserved region across different species (Figure 2(b)).

The probability of being loss-of-function (LoF) intolerant (pLI) is 0.64, and the expected number of LoF is 11.7 while the observed number is only 2, suggesting the probability of being a functionally important variant [39]. Expanded screening of *SLC16A9* in a cohort of 30 patients with gout failed to identify any additional rare variants in this gene. Taken together, WES revealed a putative causal variant in *SLC16A9* in a family with early-onset gout.

## 4. Discussion

In the present study, we recruited an unusual pedigree with early-onset gout. It is reasonable to speculate that it is possibly caused by rare monogenic variants because of two reasons, the family history and early-onset age. Firstly, this gout pedigree exhibited an autosomal dominant-like trait, consistent with a monogenic aetiology. More importantly, both proband and his affected father suffered first gout flare at early age. Epidemiological studies show that gout incidence increases with age until the age of 70 years and that onset before the age of 40 years is unusual [40, 41]. A few studies have demonstrated that complex disease with early-onset age could be caused by monogenic inheritance of mutated genes [42–44]. Therefore, aiming to investigate potential causal gene in this pedigree, we used WES which has proven to be highly robust and efficient in the identification of disease-causing genes in monogenic conditions or complex disorders [45]. Using this approach, we identified a rare missense in *SLC16A9* gene by the comprehensive analyses including allele frequency, in silico assessments, gene function, and cosegregation analysis.

A few studies supported our results of the association between *SLC16A9* and gout. The first evidence reported by Kolz et al. observed a SNP in *SLC16A9*, rs12356193, was significantly associated with serum uric acid levels by a meta-analysis of 28,141 individuals of European descent (*P*=1.1 × 10^−8^) [21]. Then the locus was successfully replicated in a cohort of 7,795 individuals [22]. Nakayama et al. investigated the relationship between another common variant (rs2242206) and gout. They found that the *P* value was significant in renal overload gout (ROL), but not with all gout susceptibility [46]. Subsequently, Köttgen et al. confirmed the *SLC16A9* locus was associated with serum urate concentrations (rs1171614, *P*=2.3 × 10^−28^), but showed only nominal association with gout (rs1171614, *P*=1.7 × 10^−2^) [7]. Phipps-Green et al. tested 28 loci for association with gout in 1536 cases with gout and 2645 controls. At *SLC16A9*, the observed association with gout was restricted to the lower Polynesian ancestry group (rs12356193, *P*=0.006) [11]. Of note, the relationship between GWAS signals and genes underlying Mendelian phenotypes has been observed [47, 48]. Thus, it is reasonable to find rare pathogenic variants in GWAS signals. In addition to these genetic association studies, several studies also provided functional evidence. *SLC16A9* is ubiquitously expressed and is especially expressed at a high level in the kidney [19, 20]. *ALDH16A1* gene is associated with serum uric acid levels and gout, and RNA sequencing in the kidney of wild-type (WT) and Aldh16a1 knockout (KO) mice revealed changes in *Slc16a9* are localized to the apical membrane of the proximal convoluted tubule cells and influence uric acid homeostasis [49]. These findings suggested the potential role of *SLC16A9* in the aetiology of gout.

However, there are two main limitations in this study. Firstly, no functional genomics studies were performed in the present study. Experimental validations are essential to determine if interesting variants are indeed responsible for clinical symptoms [50, 51]. For example, a recent study demonstrates the rare variants of ABCG2 at both the clinical level and the functional level by complex approach [52]. Second is the lack of independent replication family. The genetic screening of *SLC16A9* in gout pedigrees is required in the future studies. The copy number variations (CNVs) are not considered in this study [53].

In conclusion, we provide the first evidence for the association of rare missense in *SLC16A9* with early-onset gout. These findings not only expand our current understanding of gout, but also may have further implications for the treatment and prevention of gout.

## Figures and Tables

**Table 1:** Summary of clinical observations of the participants in this study.

ID	Gender	Age (y)	SUA	HUA	SCr	BUN	FEUa	UPH	Onset age (y)	Arthritis	Tophi	TG	TC	Obesity	HBP	HG	Obesity
II:1	F	63	257	−	51	6.8		6.5	−	−	−	−	−	−	+	−	−
II:4	M	48	581	+	69	4.2	3.77	6.0	25	+	−	−	+	+	+	+	+
II:5	F	46	305	−	44	6.0		5.5	−	−	−	+	−	+	−	−	+
III:1	M	45	321	−	50	5.3		6.0	−	−	−	−	−	−	−	−	−
III:2	M	25	517	+	71	5.1	4.63	5.0	20	+	−	−	−	−	−	−	−

SUA, serum uric acid, *μ*mol/l; HUA, hyperuricemia; SCr, serum creatine, *μ*mol/l; BUN, blood urea nitrogen, mmol/l; FEUa, fractional excretion of uric acid,%; (hyperuricemia: male > 420 *μ*mol/l; female > 360 *μ*mol/l); UPH, urine PH; TG, triglyceride; TC, total cholesterol; HBP, high blood pressure; HG, hyperglycemia.

**Table 2:** Variant identified in patients with early-onset gout.

ID	Variant	Type	Frequency (allele count)				In silico assessments			
			gnomAD	ExAC	ESP	1K	Polyphen-2	MutationTaster	LRT	M-CAP
II:4	c.277C>A, p.L93M	Hetero	0.0032% (8)	0.0033% (4)	0	0.04% (2)	Damaging	Damaging	Damaging	Possibly pathogenic
III:2	c.277C>A, p.L93M	Hetero	0.0032% (8)	0.0033% (4)	0	0.04% (2)	Damaging	Damaging	Damaging	Possibly pathogenic

## Data Availability

Summary data are available from the corresponding author on request.
